# STAT3 Is an Upstream Regulator of *Granzyme G* in the Maternal-To-Zygotic Transition of Mouse Embryos

**DOI:** 10.3390/ijms22010460

**Published:** 2021-01-05

**Authors:** Huan Ou-Yang, Shinn-Chih Wu, Li-Ying Sung, Shiao-Hsuan Yang, Shang-Hsun Yang, Kowit-Yu Chong, Chuan-Mu Chen

**Affiliations:** 1 Department of Life Sciences, and Ph.D. Program in Translational Medicine, National Chung Hsing University, Taichung 402, Taiwan; huan4096052151@alumni.nchu.edu.tw (H.O.-Y.); 178241@cch.org.tw (S.-H.Y.); 2 Department of Animal Science and Technology, National Taiwan University, Taipei 106, Taiwan; scw01@ntu.edu.tw; 3 Institute of Biotechnology, National Taiwan University, Taipei 106, Taiwan; liyingsung@ntu.edu.tw; 4 Reproductive Medicine Center, Department of Gynecology, Changhua Christian Hospital, Changhua 515, Taiwan; 5 Department of Physiology, National Cheng Kung University, Tainan 70101, Taiwan; syang@mail.ncku.edu.tw; 6 Institute of Basic Medical Sciences, National Cheng Kung University, Tainan 70101, Taiwan; 7 Department of Medical Biotechnology and Laboratory Science, College of Medicine, Chang Gung University, Taoyuan 333, Taiwan; kchong@mail.cgu.edu.tw; 8 Department of Laboratory Medicine, Chang Gung Memorial Hospital, Linkou, Taoyuan 333, Taiwan; 9 The iEGG and Animal Biotechnology Center, and Rong-Hsing Translational Medicine Research Center, National Chung Hsing University, Taichung 402, Taiwan

**Keywords:** STAT3, granzyme g, preimplantation embryo, maternal-to-zygotic transition

## Abstract

The maternal-to-zygotic transition (MZT), which controls maternal signaling to synthesize zygotic gene products, promotes the preimplantation development of mouse zygotes to the two-cell stage. Our previous study reported that mouse granzyme g (Gzmg), a serine-type protease, is required for the MZT. In this study, we further identified the maternal factors that regulate the *Gzmg* promoter activity in the zygote to the two-cell stage of mouse embryos. A full-length *Gzmg* promoter from mouse genomic DNA, FL-p*Gzmg* (−1696~+28 nt), was cloned, and four deletion constructs of this *Gzmg* promoter, Δ1-p*Gzmg* (−1369~+28 nt), Δ2-p*Gzmg* (−939~+28 nt), Δ3-p*Gzmg* (−711~+28 nt) and Δ4-p*Gzmg* (−417~+28 nt), were subsequently generated. Different-sized *Gzmg* promoters were used to perform promoter assays of mouse zygotes and two-cell stage embryos. The results showed that Δ4-p*Gzmg* promoted the highest expression level of the enhanced green fluorescent protein (EGFP) reporter in the zygotes and two-cell embryos. The data suggested that time-specific transcription factors upregulated *Gzmg* by binding cis-elements in the −417~+28-nt *Gzmg* promoter region. According to the results of the promoter assay, the transcription factor binding sites were predicted and analyzed with the JASPAR database, and two transcription factors, signal transducer and activator of transcription 3 (STAT3) and GA-binding protein alpha (GABPα), were identified. Furthermore, STAT3 and GABPα are expressed and located in zygote pronuclei and two-cell nuclei were confirmed by immunofluorescence staining; however, only STAT3 was recruited to the mouse zygote pronuclei and two-cell nuclei injected with the Δ4-p*Gzmg* reporter construct. These data indicated that STAT3 is a maternal transcription factor and may upregulate *Gzmg* to promote the MZT. Furthermore, treatment with a STAT3 inhibitor, S3I-201, caused mouse embryonic arrest at the zygote and two-cell stages. These results suggest that STAT3, a maternal protein, is a critical transcription factor and regulates *Gzmg* transcription activity in preimplantation mouse embryos. It plays an important role in the maternal-to-zygotic transition during early embryonic development.

## 1. Introduction

During early embryogenesis, the first wave of the embryo development process is the maternal-to-zygotic transition (MZT) in which embryos are under maternal signaling control until zygotic gene activation (ZGA) [1,2,3]. MZT processing involves maternal clearance and activates a cascade of early zygotic genes. Maternal clearance involves the storage and degradation of maternal transcripts and proteins that are necessary for oocyte maturation and fertilization and constitutes the first wave of ZGA [4,5,6]. During ZGA, maternal transcripts and proteins trigger zygotic gene activation. Altogether, MZT dramatically reprograms terminally differentiated germ cells into totipotent embryos and initiates the embryo development process [1,2]. In mice, a minor wave of ZGA begins at the zygote stage, and the major ZGA wave is detected at the two-cell stage [7].

Recent reports have shown several mechanisms of maternal transcript clearance. These reports include the mechanisms by which the mRNA stability is mediated by deadenylation [5,8] and small RNAs, such as microRNAs (miRNAs), endogenous small interfering RNAs (endo-siRNAs) and Piwi-interacting RNAs (piRNAs) [9,10]. Furthermore, maternal protein degradation is also a crucial mechanism for the MZT. Mouse embryos show arrested development after treatment with MG132, a proteasome inhibitor [11]. A caspase inhibitor, zVAD-FMK, caused the lethality of mouse preimplantation embryos at the eight-cell-to-blastocyst stage. Furthermore, in mouse embryos, morula compaction is suppressed upon Caspase 8 inhibition. These reports support the supposition that proteasome is involved and Caspase 8 regulates the stability of maternal proteins.

Our previous report demonstrated that granzyme g (Gzmg), a serine protease, is precisely expressed at the mouse two-cell stage and is one of the zygotic genes activated during the first wave of expression. The inhibition of *Gzmg* mRNA or proteins by a *Gzmg* morpholino or Gzmg-specific inhibitor arrested mouse embryos at the two-cell stage. In addition, zygotic RNA synthesis is inhibited after *Gzmg* knockdown by a *Gzmg* morpholino in arrested two-cell embryos. As a result, we suggest that *Gzmg*, a first wave zygotic gene, is involved in the major ZGA wave in the two-cell stage by degrading maternal proteins [12].

Signal transducer and activator of transcription 3 (STAT3) is a transcription factor with many important functions in normal and transformed cells. STAT3 is an important regulator of normal stem cells and cancer stem cells [13]. However, STAT3 regulation is highly complex, as it is involved in many different signaling pathways. Phosphorylation-activated STAT3 is sufficient to maintain the undifferentiated state of mouse embryonic stem cells (mESCs), but there is a threshold level of this inhibition: a lower expression of constitutively active STAT3 in mESC lines is not able to fully inhibit stem cell differentiation [14]. STAT3 activation in mESCs triggers the expression of several important genes that are known regulators of pluripotency, such as *Myc* and *Bcl3* [15,16]. Activated STAT3 is also known to cooperate with Nanog, another key component of pluripotency [17]. These interactions indicate that STAT3 plays a major role in regulating mESC fate. However, the regulatory role of STAT3 in early embryonic development is still unknown.

Self-renewal and pluripotency are major characteristics of embryonic stem cells (ESCs). These processes are primarily controlled by several transcription factors, including STAT3, Oct3/4, Nanog, and other ESC-specific factors. Ets-related transcription factor GA-binding protein alpha (GABPα), which is encoded by *Gabp*α, is expressed in a variety of cell types, including embryonic stem cells, and is involved in cellular functions, such as cell cycle regulation, apoptosis and differentiation [18]. The disruption of *Gabp*α has been shown to drastically repress the proliferation of *Gabp*α-null ESCs and initiate cell death within two days [18]. GABP is also known as a nuclear respiratory factor 2 (NRF-2) and is required for the expression of key genes of the mitochondrial respiration chain [19]. In mouse embryonic fibroblasts (MEFs), GABP plays an essential and nonredundant role in mitochondrial biogenesis. Moreover, the function of GABPα in mESCs has been demonstrated, and GABPα regulates Oct3/4 expression by repressing Oct3/4 repressors [20].

Nevertheless, the critical maternal factors that activate the MZT are unknown, although the interaction of maternal clearance and ZGA components has been defined. Therefore, we focused on determining the critical maternal factors by analyzing the promoters of first wave zygotic genes. *Gzmg* was a good candidate, because it is a first wave zygotic gene and controls ZGA, as demonstrated in our previous report [12]. In this study, we generated a full-length *Gzmg* promoter and its derived deletion constructs for promoter assays and determined the specific regulatory cis-elements that mediate *Gzmg* expression via certain maternal transcription factors in zygote- and two-cell stage embryos.

## 2. Results

### 2.1. The ∆4-Gzmg Promoter May Contain Embryonic Stage-Specific Enhancers

**Figure 1:**
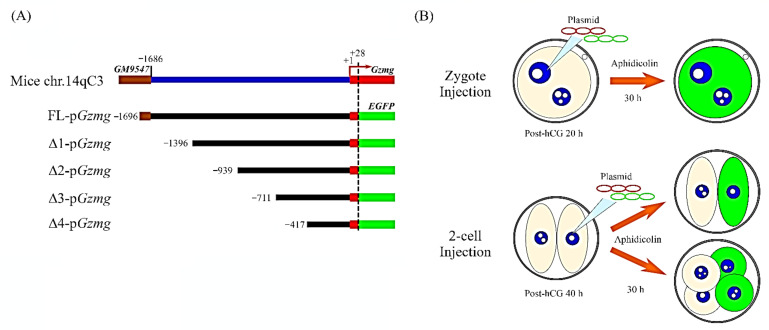
*Granzyme g* (*Gzmg*) promoter deletion constructs and the strategy of the promoter assay performed with a dual fluorescence co-injection system in mouse zygotes and 2-cell stage embryos. (**A**) The full-length *Gzmg* promoter (FL-*Gzmg*; −1724~+28 nt) was cloned from the mouse genome, and four deletion constructs of the *Gzmg* promoter were generated: Δ1-*Gzmg* (−1397~+28 ng), Δ2-*Gzmg* (−967~+28 nt), Δ3-*Gzmg* (−739~+28 nt) and Δ4-*Gzmg* (−445~+28 nt). The deletion constructs of the *Gzmg* promoter were inserted into a plasmid of enhanced green fluorescent protein-N1 (pEGFP-N1) reporter expression vector to replace the original cytomegalovirus (CMV) promoter in the plasmid. (**B**) Time-course experiments of dual fluorescence plasmids co-injected with p*Gzmg*-EGFP-N1 and mCherry control plasmid DNA in the zygote stage (20 h post-human chorionic gonadotropin (hCG)) and 2-cell stage (40 h post-hCG) embryos for the *Gzmg* promoter activity assay.

**Figure 2:**
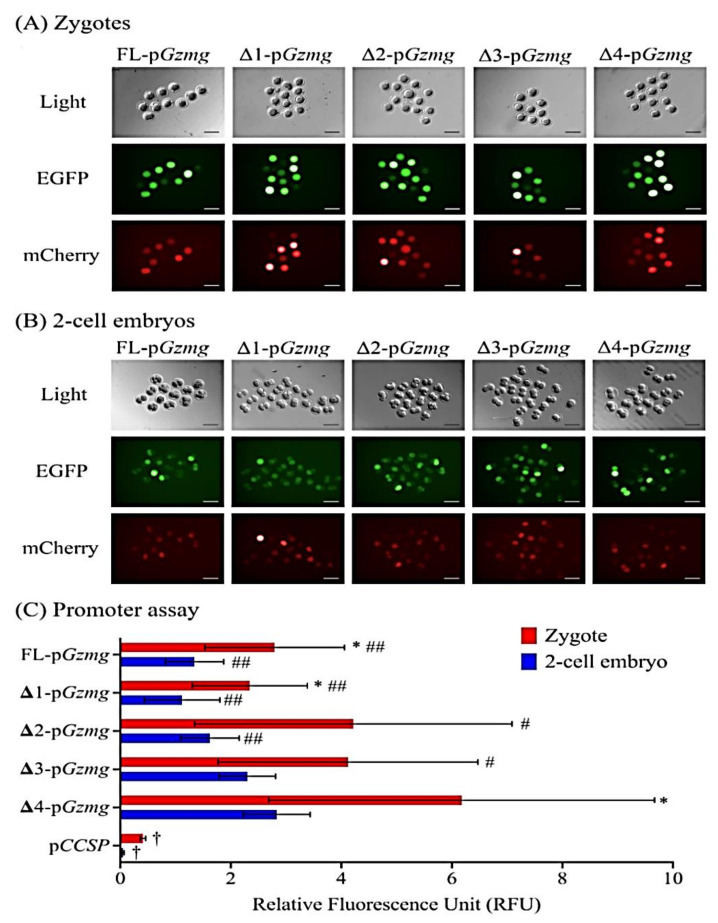
Representative images and quantitative data from the Gzmg promoter assay obtained by a dual fluorescence co-injection system in mouse zygotes and 2-cell stage embryos. (**A**) Fluorescence images from the Gzmg promoter assay of the mouse zygote stage. (**B**) Fluorescence images from the Gzmg promoter assay of the mouse 2-cell stage. Light: Bright field used to show the normal morphology of the control mouse zygotes and 2-cell stage embryos. EGFP: green fluorescence field representing the expression levels of different promoter lengths of the pGzmg-EGFP-N1 deletion constructs. mCherry: red fluorescence field representing the expression levels of co-injected mCherry control plasmid DNA. (**C**) Quantitative data of the Gzmg promoter assay obtained by the dual fluorescence system. The graph shows the means ± SD of at least four replicates for each group of embryos. The asterisk (*) indicates zygote and 2-cell stage embryo groups with the same types of Gzmg promoter constructs that show significant differences according to a Student’s *t*-test (*p* < 0.05). The number signs (^#^ and ^##^) indicate that the groups are significantly different from the Δ4-pGzmg construct groups of the same injected zygote or 2-cell stage embryos, as determined by one-way ANOVA (^#^, *p* < 0.05 and ^##^, *p* < 0.01). The dagger (^†^) indicates a nonembryonic promoter construct from the mouse lung Clara cell-specific protein (ccsp) gene promoter, used as a negative control, that shows significant differences with all types of the Gzmg promoter constructs as determined by one-way ANOVA (*p* < 0.05). RFUs: relative fluorescence intensity units.

To determine the molecular mechanism by which *Gzmg* is upregulated at the mouse two-cell stage, we investigated whether two-cell stage-specific enhancers are in the *Gzmg* promoter sequence. First, we cloned a full-length *Gzmg* promoter, FL-p*Gzmg* (−1696~+28 nt), and subsequently, we generated four deletion constructs, Δ1-p*Gzmg* (−1369~+28 nt), Δ2-p*Gzmg* (−939~+28 nt), Δ3-p*Gzmg* (−711~+28 nt) and Δ4-p*Gzmg* (−417~+28 nt), by PCR amplification. Second, these promoter constructs were ligated with an enhanced green fluorescent protein (EGFP) reporter expression vector (Figure 1A). Then, we co-injected the p*Gzmg*-EGFP-N1 constructs (250 ng/µL) and a plasmid of cytomegalovirus promoter-drived mCherry fluorescent gene (pCMV-IRES2-mCherry, 50 ng/µL), used as an internal control, into zygote pronuclei or two-cell embryonic nuclei and cultured them in a human tubal fluid (HTF) medium with a DNA synthesis (S phase) inhibitor, 4-µg/mL aphidicolin (Figure 1B). Thirty hours after injection, the different *Gzmg* promoter deletion constructs showed different expression levels of the EGFP reporter in zygotes (Figure 2A) and two-cell embryos (Figure 2B).

The zygote group injected with the Δ4-*Gzmg* promoter construct showed the highest level of EGFP reporter expression after normalization to the co-injected mCherry internal control (Figure 2C); the transcriptional activity was significantly higher than that of the other groups (relative fluorescence intensity unit (RFU) = 4.676 ± 1.031, *p* < 0.05). In the two-cell embryo-injected groups, the transcription activity of the Δ4-*Gzmg* promoter construct also showed the highest level of EGFP expression (*p* < 0.01), although the Δ3-*Gzmg* promoter construct was similarly and significantly upregulated (*p* < 0.05; Figure 2C). As a result, the zygote and two-cell embryonic stage-specific enhancers of the *Gzmg* promoter are located in the −417~+28 nt region of the Δ4-*Gzmg* promoter construct. On the other hand, the transcription activities of the FL-*Gzmg* and Δ1-*Gzmg* promoter constructs exhibited the lowest EGFP reporter expression, which might indicate that some repressors or negative regulatory sequences are located in the −1696~−939-nt *Gzmg* upstream DNA region. A nonembryonic expressed Clara cell-specific protein (CCSP) promoter construct was added to the cells in this experiment, and the results showed a significantly lower fluorescence intensity in the zygotes and two-cell embryos, and this construct served as a background control (Figure 2C).

### 2.2. Predicted Transcription Factor (TF)-Binding Sites in the Gzmg Promoter

According to the results of the promoter assay, we aimed to determine which transcription factors affect the transcriptional activity of the Gzmg promoter. To resolve this question, we predicted the TF-binding sites in the upstream Gzmg promoter by the JASPAR system. A total of 78 TF-binding sites were found in the whole Gzmg promoter. Among these sites, six TF-binding sites were found only in the −939~+28 nt Gzmg promoter region, and eight TF-binding sites were found only in the −1696~−939 nt Gzmg promoter region (Table 1). 

The Expression Atlas website was used to analyze the expression pattern of each putative TF. The transcription activities of the Hif1a, Esr2, Arnt, Sox9 and Stat3 genes were detected at the mouse oocyte and zygote stages; therefore, these transcription factors are likely maternal proteins. Moreover, mRNAs of the transcription factors Gata1, Arnt and Gabpα were found to be expressed at the mouse two-cell stage, and transcripts of Gata1, Tal1 and Evi1 were also expressed at the mouse eight-cell stage. Furthermore, in addition to being a potential maternal protein, STAT3 is expressed at the blastocyst stage. First, we focused on the GABPα, SOX9 and STAT3 TF-binding sites, which are located in the −939~+28-nt Gzmg promoter region. The transcription levels of these three TFs showed quite different patterns at each developmental stage of mouse preimplantation embryos. The expression level of the Gabpα mRNA increased at the two-cell stage and decreased at the eight-cell to blastocyst stage, and it was lethal at the preimplantation stage in the mouse embryos with the Gabpα gene knocked out. Stat3 mRNA was highly expressed at the mouse oocyte, zygote and blastocyst stages, and Stat3 knockout embryos exhibited decreased inner cell mass (ICM) proliferation (Table 1). In contrast, Sox9 mRNA was expressed only at the oocyte and zygote stages in mouse embryos, but knocking out Sox9 in the embryos caused lethality at the E11.5 stage and organogenesis defects that did not correlate with the Gzmg regulation period. Therefore, we excluded SOX9 from the subsequent analysis.

### 2.3. STAT3 Interact with the Δ4-Gzmg Promoter in the Mouse Zygotes and Two-Cell Stage Embryos

**Figure 3:**
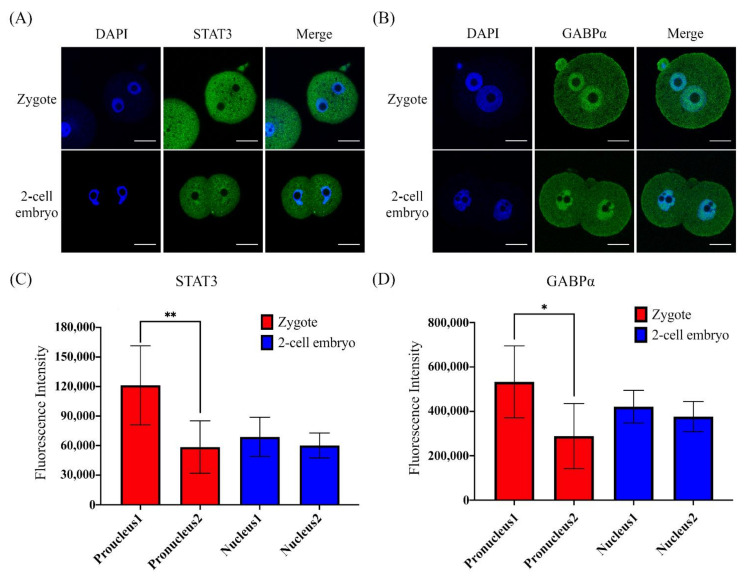
Immunofluorescence staining showing signal transducer and activator of transcription 3 (STAT3) and GA-binding protein alpha (GABPα) expression and localization in mouse zygotes and 2-cell stage embryos. Localization and expression of two transcription factors, STAT3 (**A**) and GABPα (**B**), in zygote stage embryos 20 h post-hCG and 2-cell stage embryos 40 h post-hCG. DAPI: 4′,6-diamidino-2-phenylindole, a blue-fluorescent dye that binds to AT-rich regions of double-stranded DNA and is used for nuclear localization staining. The abundance of green fluorescence representing the expression and localization of transcription factors STAT3 (zygote, *n* = 5 and 2-cell embryo, *n* = 6) and GABPα (zygote, *n* = 5 and 2-cell embryo, *n* = 6) in early-stage mouse embryos. Scale bar = 20 µm. Quantitative data of the fluorescence intensities of STAT3 (**C**) and GABPα (**D**) located in the zygotic pronuclei or 2-cell stage embryonic nuclei. The asterisk (*) indicates a significant difference between two pronuclei (*, *p* < 0.05 and **, *p* < 0.01) in each embryo as analyzed by Student’s *t*-test.

Although a previous report showed Stat3 and Gabpα mRNA expression at the mouse preimplantation stage, whether STAT3 and GABPα proteins are activated at the mouse zygote or two-cell stage was unknown. The immunofluorescence images showed that STAT3 (Figure 3A) and GABPα (Figure 3B) proteins were located at zygote pronuclei and two-cell embryonic nuclei. Furthermore, the fluorescence intensity units (FUs) of both STAT3 (*p* < 0.01; Figure 3C) and GABPα (*p* < 0.05; Figure 3D) varied between pronuclei. In the two-cell embryos, the FUs of both STAT3 and GABPα were not different between the two blastomere nuclei (Figure 3C,D). These data suggest that STAT3 and GABPα are truly expressed at the zygote stage and stably maintained in two-cell stage embryos. As a result, STAT3 and GABPα are maternal transcription factors that may reprogram pronuclei and regulate ZGA-related genes in mouse zygotes and two-cell stage embryos.

**Figure 4:**
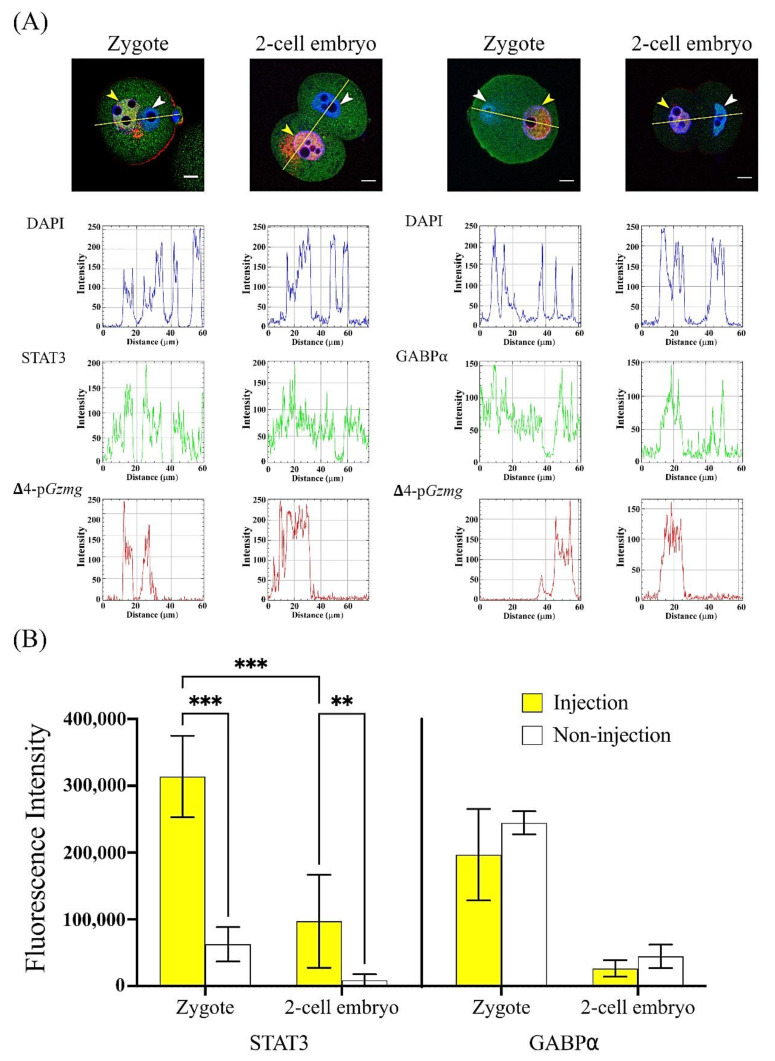
STAT3 accumulates in the pronuclei and nuclei after microinjection of the Δ4-Gzmg promoter construct plasmid in mouse zygotes and 2-cell stage embryos. (**A**) Plot profile yellow lines represent the abundance and localization of transcription factors STAT3 (left panel) and GABPα (right panel), with or without Δ4-Gzmg promoter construct plasmid injection into one side of the zygotic pronuclei and 2-cell embryonic nuclei. Yellow arrowheads indicate nuclei after Δ4-Gzmg promoter construct plasmid injection. White arrowheads indicate nuclei without Δ4-Gzmg promoter construct plasmid injection. Scale bar = 10 µm. (**B**) Quantitative data were calculated by absolute difference values of fluorescence intensities of STAT3 (zygote, *n* = 5 and 2-cell embryo, *n* = 6) and GABPα (zygote, *n* = 6 and 2-cell embryo, *n* = 5) between the Δ4-Gzmg promoter plasmid-injected nucleus and the noninjected nucleus from the same zygote or 2-cell stage embryo. The asterisks (** and ***) indicate a significant difference as determined by Student’s *t*-test. (**, *p* < 0.01 and ***, *p* < 0.001).

To test whether STAT3 and GABPα interact with the Gzmg promoter, especially in the −417~+28-nt Gzmg upstream DNA region. A Cy5-labeled Δ4-Gzmg promoter construct plasmid was injected into one pronuclei of the zygotes and one nucleus of the two-cell stage embryos. Six hours after injection, both STAT3 and GABPα colocalized with the Cy5-labeled Δ4-Gzmg promoter construct plasmid (Figure 4A). Interestingly, the absolute abundance of the STAT3 colocalized in the zygotic pronuclei of the injection group was significantly greater than that of the noninjection group (*p* < 0.01), and the same results were also observed in the two-cell stage embryos (*p* < 0.01). However, the amount of GABPα located in the nuclei was not different between the injected groups and the noninjected groups (Figure 4B). Moreover, the abundance of STAT3 colocalized in the zygote pronuclei after Δ4-Gzmg promoter construct injection was significantly higher than that in the nuclei of the two-cell stage embryos injected with the same construct (*p* < 0.05). These data indicated that the STAT3 transcription factor can be recruited into pronuclei or nuclei to interact with the −417~+28-nt Δ4-Gzmg upstream DNA region. Additionally, we deleted the putative STAT3-binding site from the Δ4-Gzmg promoter construct and called it the ΔS-pGzmg construct. After the same dose injection, the data showed that the transcription activity of the ΔS-pGzmg construct was lower than the Δ4-pGzmg construct by promoter assay (*p* < 0.001; Supplementary Figure S1). Therefore, we hypothesized that Gzmg gene transcription may be triggered by the STAT3 transcription factor.

### 2.4. STAT3 Is a Critical Factor for Promoting Early Embryonic MZT in Mice

**Figure 5:**
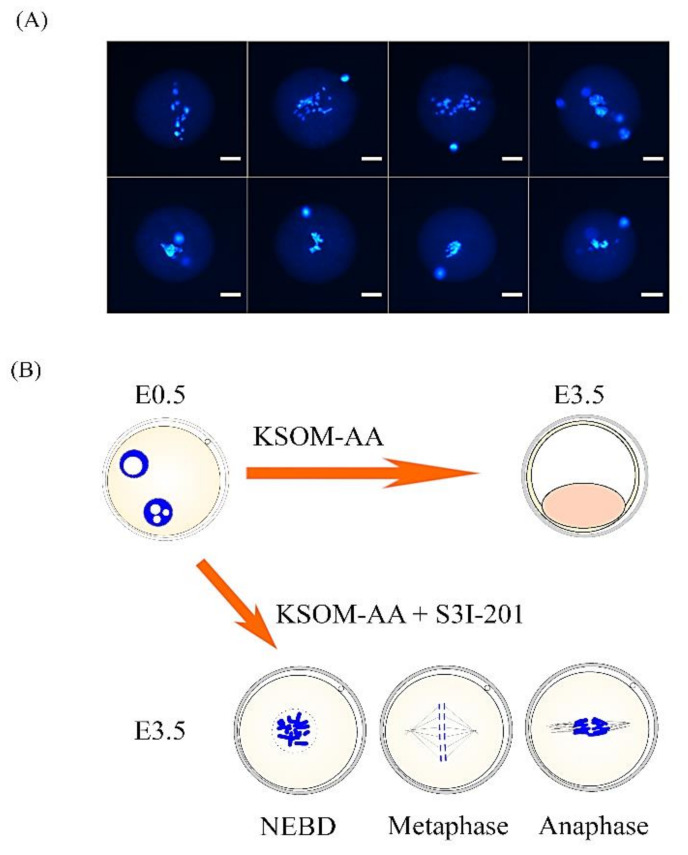
Abnormal chromosome plate in arrested zygote stage embryos treated with STAT3 inhibitor S3I-201. (**A**) Representative images of abnormal metaphase chromosome plates in arrested zygotes after treatment with 100-μM S3I-201 inhibitor in KSOM-AA embryo culture medium. Chromosomal DNA was stained with DAPI, a blue fluorescent dye. All samples were analyzed under a laser scanning confocal microscope. Scale bar = 20 µm. (**B**) Representative images of the different morphologies of the abnormal metaphase chromosome plates.

We next sought to investigate whether maternal STAT3 can trigger MZT at the mouse zygote and two-cell stages. Two STAT3 inhibitors, S3I-201 and WP1066, were used to analyze the success rate of mouse embryos in different preimplantation development stages (Table 2). Zygotes were cocultured with STAT3 inhibitors or dimethyl sulfoxide (DMSO) solvent in KSOM-AA medium at 22 h post-human chorionic gonadotropin (hCG) treatment. An untreated control group (KSOM-AA) was added to this experiment as a normal embryonic culture reference. The control groups developed normally to the blastocyst stage (KSOM-AA, 95%, *n* = 59 and DMSO, 89%, *n* = 56). In the WP1066-treated group (*n* = 57), 31.6% of the embryos arrested at the two-cell stage and 68% of the embryos developed into blastocysts. The majority of the embryos cocultured with S3I-201, a more specific STAT3 inhibitor, arrested between the zygote and two-cell stages (34.1% and 65.9%, respectively; *n* = 44). The transcription activity of the Gzmg promoter was significantly decreased after being treated with S3I-201 (*p* < 0.05) (Table 2). Furthermore, we also found that one-cell embryos treated with S3I-201 mostly arrested at the M phase (Figure 5A) and showed differing abnormal chromosome plate morphologies (Figure 5B). These results suggest that blocking STAT3 function by specific inhibitors resulted in dramatic arrest during the MZT to block normal development before the blastocyst stage in an in vitro culture system.

### 2.5. A Novel STAT3 Isoform May Be Expressed in Mouse Zygotes and Two-Cell Stage Embryos

**Figure 6:**
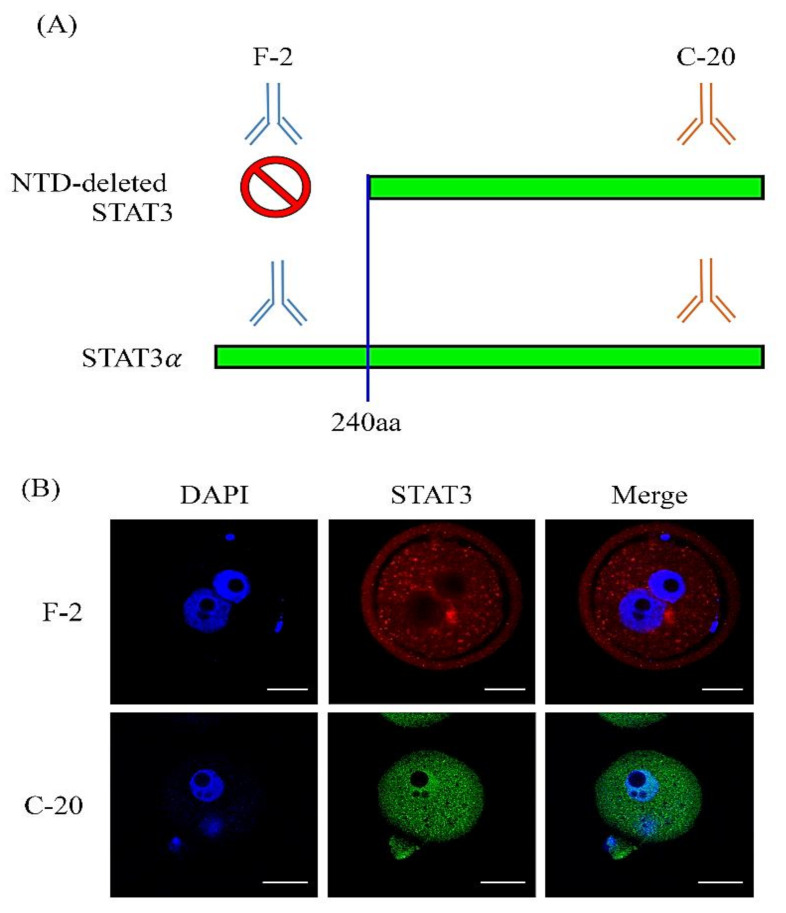
A novel N-terminal domain (NTD)-deleted STAT3 in the pronucleus of mouse zygote stage embryos. (**A**) A graphic hypothesis showing that the N-terminal domain (NTD) deletion of STAT3 may be detected by two different recognition domains of STAT3 antibodies. One is labeled with an anti-F-2 antibody that specifically recognizes the N-terminal domain of STAT3 and is used for full-length STAT3α detection. The other is an anti-C-20 antibody that specifically recognizes the C-terminal domain of STAT3 and is used for the detection of both NTD-deleted STAT3 and STAT3α. (**B**) Immunolocalization of NTD-deleted STAT3 and full-length STAT3α in mouse zygote stage embryos by anti-F-2 (red fluorescent color) and anti-C-20 (green fluorescent color) antibodies. DNA was stained with DAPI (blue fluorescent color) to define pronuclei localization. Scale bar = 10 µm.

A previous report showed that the STAT3 protein is not located in the nuclei at the mouse zygote or two-cell stage [34], which is quite different from our data. Therefore, we hypothesized that a novel STAT3 isoform exists in mouse zygotes and two-cell stage embryos. Two different monoclonal antibodies recognizing different epitopes of the STAT3 protein were used in this study: an anti-F-2 antibody for the N-terminal domain and an anti-C-20 antibody for the C-terminal domain of STAT3 recognition (Figure 6A). As expected, we found a novel isoform in the N-terminal domain (NTD)-deleted STAT3 protein that exhibited abundant signals in the pronuclei of the zygotes when the anti-C-20 antibody was used for the whole-mount in situ immunofluorescence analysis (Figure 6B).

## 3. Discussion

In this study, we successfully used a promoter assay to quantify the transcription activities of different regions of *Gzmg* upstream promoters in mouse zygotes and two-cell stage embryos. Fourteen transcription factors were predicted to be candidates for key maternal factors in the *Gzmg* promoter region. Then, we focused on transcription factors that have been detected at mouse preimplantation embryo stages and have been found to affect embryo development, such as STAT3 and GABPα. The immunofluorescence results showed that both GABPα and STAT3 were localized in zygote pronuclei and two-cell embryo nuclei. These data suggest that both GABPα and STAT3 may regulate the transcriptional activities of zygotic genes at the mouse zygote and two-cell stages.

It is known that embryos cultured in 4-µg/mL aphidicolin can arrest their development at the beginning of the S phase and easily control the timing of gene expressions in the early embryonic stages. Since the S phase has not yet begun in one-cell embryos, one-cell embryos retained their two pronuclei throughout the experiment. However, two-cell embryos were isolated after they underwent DNA replication; they cleaved into four cells [35]. According to the reports that we followed, their evidence supports a time-dependent mechanism for the initiation of zygotic gene activation, which is not prohibited by aphidicolin treatment [35,36,37,38,39]. Therefore, we did not directly connect the embryo morphologies and expression patterns of the embryo stages in this study.

In the promoter assay, the transcription activity of *Gzmg* upstream promoters in the zygote stage is more potent than in the two-cell stage embryos. It seems to not intuitively coincide with our previous report [12]. The initiation period of ZGA is the way to explain why the expression level of the *Gzmg* reporter construct in the zygote group was higher than in the two-cell stage embryo group. The DNA injection time was before the initiation of ZGA in the zygote group. However, the DNA injection time of the two-cell embryo group was after the initiation of ZGA. The time of observations were at 30 h post-injection in both groups. Thus, the actual expression periods of *Gzmg* reporters are at the zygote to the two-cell stage in the zygote group and at the two-cell to the four-cell stage in the two-cell group under aphidicolin treatment. A previous report supports that the embryonic gene expression period at two-cell stage embryos may be from the onset of ZGA to the time before the four-cell stage. Consequently, the difference between the two groups is probably evidence that the *Gzmg* promoter is activated in a time-dependent manner [36].

In *Gabp*α-knockout mice, embryos were not detected during implantation; therefore, *Gabp*α^−/−^ embryos may undergo developmental failure at the preimplantation embryo stage [30]. GABPα is a key factor in the self-renewal and differentiation of hematopoietic stem cells. Furthermore, GABPα is important for disrupting neuromuscular junction synaptic function [40] and is also required for cell cycle progression [41]. Interestingly, GABPα can regulate interleukin 17 receptor alpha (IL-17Rα) in cytotoxic T cells [42], and *Gzmg* is also expressed in cytotoxic T cells (CTLs) [43]. Therefore, *Gzmg* might be upregulated by GABPα at the mouse zygote and two-cell stages. On the other hand, GABPα acts as a regulator of B lymphocyte development [44]. Although GABPα was not recruited by the Δ4-*Gzmg* promoter at the zygote and two-cell stages, GABPα might regulate the transcriptional activity of *Gzmg* by the CTL-related pathway according to its ability to regulate gene expression in CTLs and B lymphocytes.

STAT3, a member of the STAT family, is phosphorylated by Janus kinases (JAKs), which are nonreceptor tyrosine kinases (nRTKs). Then, phosphorylated STAT3 forms homo- or heterodimers and is translocated into nuclei where activated STAT3 dimers execute the role of transcription activators [45]. STAT3 can be activated by the JAK-STAT3 signaling pathway in response to certain ligands, such as growth hormone, epidermal growth factor (EGF), IL-5, IL-6, leukemia inhibitory factor (LIF) and interferons, and by cross-talk with components in the Notch signaling pathway [46]. STAT3 plays multiple functions and regulates many cellular processes, including the cell growth and apoptosis of metastatic cancer cells [47]. Mouse embryo development fails when the *Stat3* gene is disrupted, and the number of cells in the inner cell mass is decreased in *Stat3*-disrupted blastocysts [33]. Indeed, the LIF-JAK-Stat signaling pathway plays a role in the self-renewal of embryonic stem cells (ESCs). The pathway is activated by LIF, which is an IL-6 class cytokine. STAT3 is activated by JAK after LIF bind with LIF receptors to trigger JAK activity. Then, STAT3 upregulates the transcriptional activity of *Klf4*, and KLF4 activates pluripotency-related genes, such as *Oct4* and *Sox2* [48]. The LIF-JAK-Stat signaling pathway is also involved in de novo DNA methylation and epigenetic modification by activating de novo DNA methyltransferases and inhibiting histone deacetylases [49]. According to these reports, STAT3 plays the role of the somatic cell reprogramming factor in blastocysts and ESCs. In this study, we first demonstrated that STAT3 acts as an important transcription factor in early embryonic development to trigger the MZT in the mouse zygote and two-cell stages.

To analyze whether transcription factors interact with the *Gzmg* promoter, an in-situ DNA–protein interaction assay was developed. Usually, an electrophoretic mobility shift assay (EMSA) is used for detecting the binding capacity of DNA and a protein [50]. Colocalization assays usually present evidence of two-component interactions in situ, such as protein–protein interactions. Unfortunately, it is difficult to collect enough material for mouse embryos to perform an EMSA. It is also difficult to directly prove the binding capacity between the *Gzmg* promoter and STAT3. Therefore, we used a colocalization assay to observe the *Gzmg* promoter and STAT3 interaction. The *Gzmg* promoter was successfully labeled with Cy5 fluorescence by a Label IT^®^ nucleic acid labeling kit, which can be used to replace the primary antibody for detecting DNA. For our study, we used a new method for analyzing DNA–protein interactions at the mouse zygote and two-cell embryo stages.

During the onset of zygotic gene activation, only STAT3 was recruited into the pronuclei and two-cell nuclei by the Δ4-*Gzmg* promoter. The results of blocking the STAT3 protein activity showed that mouse embryo development was arrested at the zygote and two-cell stages. Although both S3I-201 and WP1066 are STAT3 inhibitors, their efficacies in embryonic development arrest were dramatically different at the zygote and two-cell stages: 100% for S3I-201 and 31.6% for WP1066. S3I-201 is a direct STAT3 inhibitor by the de-dimerization of STAT3 [51], while WP1066 is an AG490 tyrphostin analog that disrupts JAK2 protein tyrosine kinase [52]. JAK2 is crucial for signal transduction from hormone-like cytokines and some cytokines that signal through IL-3, IL-6 and gp130 receptors [53]. A previous study of *Jak2*-deficient mice showed embryonic lethality caused by erythropoiesis failure [54]. Furthermore, JAK2 acts as an important signaling transductor of Nanog regulation for ES cell self-renewal [55]. However, we found that most embryos survived to the blastocyst stage after WP1066 treatment at 22 h post-hCG. These data suggested that maternal STAT3 proteins were activated by a JAK2-independent pathway before the MZT switch.

One of the JAK2-independent pathways is known as the IL-2-JAK1,3-STAT3 signaling pathway. IL-2 widely regulates homeostasis and the immune system, especially in T-regulator cells [56]. IL-2 and IL-15 only activate the slowly migrating isoform STAT3α in human CD4^+^ T cells [57]. The C-20 antibody is known to recognize the C-terminal domain of STAT3α [58]. Furthermore, IL-2 induces both the tyrosine and serine phosphorylation of STAT3α in T lymphocytes. In CTLs, IL-2 can stimulate high levels of granzyme B expression. Granzyme genes play roles in cell-mediated cytotoxicity [59]. These proteins can also be induced in natural killer (NK) cells by IL-2 and IL-12 [60]. However, IL-12-dependent antitumor immunity is inhibited by STAT3, which activates the IL-23 signaling pathway [61]. This means that IL-12 alone might not positively regulate STAT3. During pregnancy, granulated metrial gland (GMG) cells, which are NK cells, proliferate and differentiate in the murine uterus. *Granzyme* genes are upregulated by IL-2 and IL-15 in GMG cells from days nine to 17 of gestation [12,62]. In addition, IL-2 was found by a multiplex proteome analysis in human follicle fluid, and the lower level of IL-2 in follicle fluid correlated with the inefficient in vitro fertilization (IVF) cycle [63]. IL-15 also affects oocyte maturation in follicular fluid [64]. Taken together, IL-2 and IL-15 positively regulate *Stat3* and *granzyme* genes. STAT3 may act as a transcriptional regulator to interact with the *Gzmg* promoter found in this study. This evidence suggests that Granzyme g protein expression might be upregulated by JAK2-independent pathways, such as IL-2 and IL-15/JAK1,3/STAT3 signaling.

In *Stat3^fl/fl^*; *Zp3-Cre* transgenic mice, oocytes are conditionally deleted by *Stat3* at the primary follicular stage. The *Stat3*-deleted oocytes showed normal maturation, fertilization and preimplantation development [65]. The current study also showed that the oogenesis was normal when maternal *Stat3* was deleted at the primordial follicular stage in *Stat3*^*f*/*f* (*f*/−)^; *Gdf9*-*iCre* female mice [66]. Therefore, STAT3 may be expressed before the primordial follicular stages or have other origins, such as cumulus cells [67,68]. In addition, STAT3 inhibitors, Stattic and BP-1-102 were used to analyze the correlation between STAT3 phosphorylation and oocyte maturation. Interestingly, Y705-pSTAT3 plays a novel role of spindle assembly at oocyte maturation [66]. Those reports indicate that STAT3 plays multiple roles at oogenesis and preimplantation development. In the zygote and two-cell embryo stages, STAT3 may interact with the spindle assembly in our inhibitor assay. Nevertheless, Y705-pSTAT3 was not detectable from germinal vesicle breakdown (GVBD) to early two-cell stage embryos [34,66]. STAT3 is not possible to play the role of spindle assembly by Y705 phosphorylation. Thus, it remains largely unknown, which is the mechanism of STAT3 in zygote and two-cell stage embryos.

A previous study demonstrated that maternal STAT3 is expressed at the full preimplantation embryo stages, but this protein is not expressed in the zygote pronuclei or two-cell nuclei [34]. Interestingly, our data showed that maternal STAT3 proteins exist and interact with the *Gzmg* promoter in the zygote pronuclei and two-cell nuclei. The opposite results between these two studies are because different domains of STAT3 splicing variants are recognized by different antibodies. The anti-H-190 and anti-F-2 STAT3 antibodies recognize the STAT3 N-terminal peptides, but the anti-C-20 antibody recognizes the STAT3 C-terminal peptides. Our data show that maternal STAT3 proteins include two isoforms in fertilized eggs and two-cell stage embryos. One of the N-terminal truncated STAT3 interacts with chromatin in mouse zygotes and two-cell stage embryos to trigger *Gzmg* promoter activity, but the full-length STAT3α isoform has not been found to exhibit this function. However, the functions of N-terminal truncated STAT3 remain largely unknown. In general, the N-terminal domain (NTD) facilitates STAT3 dimerization or tetramerization [69,70]. Some of the transcriptional activity of genes is affected when the STAT3 N-terminus is truncated, but some genes are not affected [71]. The NTD plays a crucial role in unphosphorylated STAT3 dimerization. Interestingly, in contrast to the unphosphorylated STAT3 dimer, the phosphorylated STAT3 dimer cannot be imported into or accumulate in the nuclei without the NTD [72,73]. Thus, STAT3 may promote mouse zygote and two-cell development via unphosphorylated STAT3 dimers with truncated N-termini.

## 4. Materials and Methods

### 4.1. Isolation of the Gzmg Promoter and Deletion Construct Cloning

Mouse *granzyme g* (*Gzmg*) gene sequences, located on mouse chromosome 14qC3, were identified in the mm9 mouse genomic DNA sequence database in the National Center for Biotechnology Information (NCBI). A full-length *Gzmg* promoter from mouse genomic DNA, named FL-p*Gzmg* (−1696~+28 nt), was amplified and cloned by polymerase chain reaction (PCR) using high-fidelity Pfu *Taq* polymerase (Geneaid Biotech Ltd., Taipei, Taiwan). Subsequently, four deletion constructs of the *Gzmg* promoter, Δ1-p*Gzmg* (−1369~+28 nt), Δ2-p*Gzmg* (−939~+28 nt), Δ3-p*Gzmg* (−711~+28 nt) and Δ4-p*Gzmg* (−417~+28 nt), were generated by PCR using different 5′-upstream and the same 3′-downstream primers (Supplementary Table S1). These *Gzmg* promoter constructs were then inserted into a pEGFP-N1 expression vector to replace the original CMV promoter, as shown in Figure 1A. 

### 4.2. Mouse Zygote Collection

Zygotes were obtained from the oviducts of 6- to 8-week-old ICR female mice, as described previously [12]. Briefly, female mice were induced to superovulate by intraperitoneal injection of 10 international units (IU) of pregnant mare serum gonadotropin (PMSG, cat. no. G4877; Sigma, St. Louis, MO, USA) and, 48 h later, by injection of 10 IU of human chorionic gonadotropin (HCG, cat. no. C1063; Sigma, St. Louis, MO, USA) and mating with male mice. Zygotes were recovered in M2 medium (cat. no. M7167; Sigma, St. Louis, MO, USA) 20 h after hCG injection, and the cumulus cells were removed with 0.5-mg/mL hyaluronidase (cat. no. H4272; Sigma, St. Louis, MO, USA). Thereafter, the zygotes were cultured in human tubule fluid medium (HTF; Irvine Scientific, Santa Ana, CA, USA) in a 37 °C incubator under 5% CO^2^ [12]. The animal use protocol in this study was reviewed and approved by the Institutional Animal Care and Use Committee of the National Chung Hsing University (IACUC approval number: 96–52; approval date: 1 August 2017).

### 4.3. Promoter Assay in the Mouse Preimplantation Stage

The protocol for the promoter assay used in the mouse preimplantation stage was modified from previous reports [35,36,37]. Groups of 10~20 embryos at a time were microinjected with approximately 2 pl of supercoil plasmid DNA (p*Gzmg*-EGFP-N1 plasmid 250 ng/µL and pCMV-IRES2-mCherry 50 ng/µL) into one of the pronuclei or 2-cell nuclei using an automated microinjection system (HDJ-M3 microinjector; Prime Tech Ltd., Ibaraki, Japan) and inverted differential interference contrast microscopy (an AxioVert 135 microscope; ZEISS, Oberkochen, Germany). Surviving embryos were cultured in HTF medium with 4-μg/mL aphidicolin (cat. no. A0781; Sigma, St. Louis, MO, USA) at 37 °C in an incubator with 5% CO^2^. Thirty hours after plasmid injection, images were captured, and the fluorescence intensity of each embryo experiment was quantified by ImageJ software (Version 1.51f, National Institutes of Health, Bethesda, MD, USA) (Figure 2). A sum of approximately 10 embryos was used per experiment, and each experiment was repeated in every group with 4 replicates. Furthermore, the EGFP fluorescence intensity relative to the mCherry fluorescence was calculated as shown in Figure 2C.

### 4.4. Transcription Factor-Binding Site Prediction

All transcription factor matrix models of the JASPAR core vertebrate database (http://jaspardev.genereg.net/), were selected for analyzing the *Gzmg* promoter (accessed on 10 October 2018). The relative profile score threshold was set at 85%. Data were output after calculation through the JASPAR website. Furthermore, we chose a microarray database of mouse preimplantation embryos from the Expression Atlas website (http://www.ebi.ac.uk/gxa/home) to determine the expression time of the putative transcription factors (accessed on 10 October 2018).

### 4.5. Whole-Mount Embryo Immunofluorescence

The protocol of whole-mount embryo immunofluorescence staining in the mouse preimplantation stage was described in our previous report [12]. Briefly, mouse preimplantation embryos were removed from the zona pellucida and placed in the acid Tyrode’s solution (Sigma, St. Louis, MO, USA) for 10 sec at room temperature (RT). Then, the embryos were fixed with 4% paraformaldehyde solution for 30 min at RT. Subsequently, embryos were permeabilized with 2% Triton X-100 in phosphate-buffered saline (PBS) for 30 min at RT, blocked in 4% bovine serum albumin (BSA) in PBS and incubated overnight with anti-STAT3 antibodies C-20 (cat. no. sc-482; Santa Cruz Biotech. Inc., Santa Cruz, CA, USA) and F-2 (cat. no. sc-8019; Santa Cruz Biotech. Inc., Santa Cruz, CA, USA) or anti-GABPα antibody (cat. no. sc-22810; Santa Cruz Biotech. Inc., Santa Cruz, CA, USA) at 4 °C. After washing in PBS with 1% BSA and 0.005% Triton X-100, the embryos were incubated with donkey polyclonal secondary antibody to rabbit immunoglobulin G-heavy chain and light chain (IgG—H&L) (Alexa Fluor^®^ 488) (A-21206; Thermo Fisher Scientific Inc., Waltham, MA, USA) or donkey polyclonal secondary antibody to mouse IgG—H&L (Alexa Fluor^®^ 546) (A-10036; Thermo Fisher Scientific Inc., Waltham, MA, USA) and DAPI (4′,6-diamidino-2-phenylindole; Thermo Fisher Scientific Inc., Waltham, MA, USA) blue fluorescent DNA stain. After washing, the stained embryos were mounting by ProLong^TM^ Gold antifade reagent (P36930; Invitrogen, Carlsbad, CA, USA). Finally, the embryos were observed and imaged by laser scanning confocal microscopy (LSM 510; Carl Zeiss, Oberkochen, Germany) [12]. To test the F-2 antibody specificity, immunofluorescence staining of A549 cells and Western blot analysis of National Institutes of Health Swiss mouse embryo-3T3 (NIH/3T3) cells were used to demonstrate that the F-2 monoclonal antibody really recognizes STAT3 in the cell and total cellular protein, as shown in Supplementary Figure S2A,B, respectively.

### 4.6. In Situ DNA–Protein Interaction Assay

In this experiment, DNA of the Δ4-*Gzmg* promoter construct was first labeled with Cy5 dye by a Label IT^®^ nucleic acid labeling kit (Cy™5, cat. no. MR-MIR3725; Mirus Bio LLC, Madison, WI, USA). Next, Cy5-labeled DNA was injected into pronuclei or 2-cell nuclei in mouse embryos. Six hours after DNA injection, the injected embryos were fixed for immunostaining for detecting the STAT3 or GABPα signals in the Cy5-labeled Δ4-*Gzmg* promoter. A laser scanning confocal microscope (Carl Zeiss LSM 510) was used to analyze the localization of the DNA and proteins. Finally, a plot profile and fluorescence quantification data were used to analyze the interaction of the DNA and proteins by an ImageJ system. 

### 4.7. Inhibitor Assay

The STAT3 inhibitor assay protocol was described previously [51,74]. Briefly, embryos were exposed to inhibitors beginning at the zygote stage. The STAT3 inhibitors, 100-μM S3I201 (cat. no. sc-204304; Santa Cruz Biotech. Inc., Santa Cruz, CA, USA) [51] or 5-μM WP1066 (cat. no. ALX-270-483; Enzo Life Science Inc., New York, NY, USA) [74], and KSOM-AA medium (cat. no. MR-106; Sigma-Aldrich, St. Louis, MO, USA) were changed every 24 h. Embryos were observed by light microscopy every day to determine the developmental stages of the preimplantation embryos.

### 4.8. Quantification of Immunofluorescence

ImageJ software was used to quantify the fluorescence intensities of STAT 3 or GABPα in the zygote pronuclei and the 2-cell embryonic nuclei. We set measurements of the area, mean gray value and integrated density, which, in the fluorescence intensity, were for collecting data. Then, the target regions and three background regions from the same image were collected. The fluorescence intensity of the target regions subtracted from the intensity of an average of three background intensities times the size of the target region.

### 4.9. Statistical Analysis

All results were expressed as the means ± SEM (standard error of the mean). The data were analyzed by one-way ANOVA and lysergide (LSD) tests using SPSS software (Version 20, IBM Corp., Armonk, NY, USA). The statistical analysis was performed using Student’s *t*-test. *p* < 0.05 was considered statistically significant; *, *p* < 0.05; **, *p* < 0.01 and ***, *p* < 0.001. In addition, higher and lower fluorescence intensities of STAT3 or GABPα in the zygote pronuclei and 2-cell embryonic nuclei were defined as “pronucleus 1” and “pronucleus 2” or “nucleus 1” and “nucleus 2”, respectively, as shown in Figure 3. Absolute difference values of the fluorescence intensities between the Δ4-*Gzmg* promoter plasmid-injected nucleus and the noninjected nucleus from the same zygote or 2-cell embryo were used for the statistical analysis in Figure 4.

## 5. Conclusions

Our results suggest that STAT3, a maternal protein, is a critical transcription factor and regulates *Gzmg* transcription activity in preimplantation mouse embryos. Furthermore, a new N-terminal truncated STAT3 isoform was identified in the nucleus of the zygotes and two-cell stage embryos, which may play an important role in upregulating *Gzmg* expression to promote the maternal-to-zygotic transition during early-stage mouse embryo development.

## Figures and Tables

**Table 1:** Phenotypes after transcription factor knockout during mouse embryo development. ICM: inner cell mass and *Gzmg* (*Granzyme G*).

Promoter Region	Transcription Factor	Expression Stage in Preimplantation Embryo	Phenotype in Knock-Out Mice	Reference
*Gzmg* −1696~−939	Hypoxia-inducible factor 1-alpha (*Hif1a*)	Oocyte and zygote	Cardiac and vascular malformations and lethality at embryonic stage 10.5 days (E10.5)	[21]
	GATA-binding factor 1 (*Gata1*)	2-cell and 8-cell	Embryonic lethality from severe anemia at E10.5	[22]
	Estrogen receptor 2 (*Esr2*)	Oocyte and zygote	Reproductive system defect	[23]
	T-cell acute leukemia protein 1 (*Tal1*)	8-cell	Absence of blood formation	[24]
	Arylhydrocarbon receptor nuclear translocator (*Arnt*)	Oocyte, zygote/2-cell	Embryonic lethality from placental defect at E10.5	[25]
	Ecotropic viral integration site-1 (*Evi1*)	8-cell	Embryonic lethality at E10.5	[26]
	TEA domain transcription factor 1 (*Tead1*)	All stages	Morphogenesis defect at E9.5	[27]
	Estrogen receptor 1 (*Esr1*)	All stages	Reproductive system defect	[23]
*Gzmg* −939~+28	FEV transcription factor (*Fev*)	All stages	Respiratory dysfunction	[28]
	Forkhead box F2 (*Foxf2*)	Not detected	Cleft palate and abnormal tongue	[29]
	GA-binding protein alpha (*Gabp*α)	2-cell	Preimplantation embryo lethality	[30]
	Myogenic factor (*Myf*)	Not detected	Skeletal myogenesis defect	[31]
	SRY-box transcription factor 9 (*Sox9*)	Oocyte and zygote	Organogenesis defect at E11.5	[32]
	Signal transducer and activator of transcription 3 (*Stat3*)	Oocyte, zygote, morula and blastocyst	Decreased ICM proliferation	[33]

**Table 2:** Development rates of mouse embryos at E3.5 and transcription activities of the *Gzmg* promoter by culturing with STAT3 inhibitors.

Different Inhibitor Treatment	Total Embryos	No. (%) of Embryos Developed to						Transcription Activity (Relative Fluorescence Unit)			
		Zygote	2-Cell Embryo	4-Cell Embryo	Morula Embryo	Blastocyst Embryo	Fragment Embryo	Mean	S.D.	*p*-Value	*p*-Value
										(w/DMSO)	(w/KSOM)
S3I-201 (100 µM)	44	15 (34.1)	29 (65.9)	0 (0.0)	0 (0.0)	0 (0.0)	0 (0.0)	3.193	0.788	0.0552	0.0372
WP1066 (5 µM)	57	0 (0.0)	18 (31.6)	0 (0.0)	0 (0.0)	39 (68.4)	0 (0.0)	3.746	1.14	0.1283	0.0967
DMSO ^1^ (1/1000)	56	0 (0.0)	1 (1.8)	0 (0.0)	0 (0.0)	50 (89.3)	5 (8.9)	5.628	1.722	-	0.9921
KSOM ^2^ (Control)	59	0 (0.0)	2 (3.4)	0 (0.0)	0 (0.0)	56 (94.9)	1 (1.7)	5.639	2.158	0.9921	-

^1^ DMSO: dimethyl sulfoxide; ^2^ KSOM: KSOM-AA culture medium.

## Data Availability

The data presented in this study are available on request from the corresponding author.

## Supplementary Materials

The following are available online at https://www.mdpi.com/1422-0067/22/1/460/s1: Table S1: The designed primer information for PCR amplification of the *granzyme g* promoter deletion constructs in this study. Figure S1: The transcription activity of the *Gzmg* promoter assay between Δ4-p*Gzmg* and ΔS-p*Gzmg* in the mouse zygote stage embryos. Figure S2: Specificity test of the F-2 monoclonal antibody in the recognition of the STAT3 protein.

## Abbreviations

EMSA	electrophoretic mobility shift assay
GABPα	GA-binding protein alpha
Gzmg	granzyme g
HTF	human tubal fluid
MEFs	mouse embryonic fibroblasts
mESCs	mouse embryonic stem cells
MZT	maternal-to-zygotic transition
NTD	N-terminal domain
STAT3	signal transducer and activator of transcription 3
ZGA	zygotic gene activation

## References

[1] Li L., Lu X., Dean J. (2013). The maternal to zygotic transition in mammals. Mol. Asp. Med..

[2] Lee M.T., Bonneau A.R., Giraldez A.J. (2014). Zygotic genome activation during the maternal-to-zygotic transition. Annu. Rev. Cell Dev. Biol..

[3] Simonelig M. (2012). Maternal-to-zygotic transition: Soma versus germline. Genome Biol..

[4] Ma J., Flemr M., Strnad H., Svoboda P., Schultz R.M. (2013). Maternally recruited DCP1A and DCP2 contribute to messenger RNA degradation during oocyte maturation and genome activation in mouse. Biol. Reprod..

[5] Barckmann B., Simonelig M. (2013). Control of maternal mRNA stability in germ cells and early embryos. Biochim. Biophys. Acta.

[6] Walser C.B., Lipshitz H.D. (2011). Transcript clearance during the maternal-to-zygotic transition. Curr. Opin. Genet. Dev..

[7] Schultz R. (1993). Regulation of zygotic gene activation in the mouse. Bioessays.

[8] Lund E., Liu M., Hartley R.S., Sheets M.D., Dahlberg J.E. (2009). Deadenylation of maternal mRNAs mediated by miR-427 in *Xenopus laevis* embryos. RNA.

[9] Ohnishi Y., Totoki Y., Toyoda A., Watanabe T., Yamamoto Y., Tokunaga K., Sakaki Y., Sasaki H., Hohjoh H. (2010). Small RNA class transition from siRNA/piRNA to miRNA during pre-implantation mouse development. Nucleic Acids Res..

[10] Svoboda P., Flemr M. (2010). The role of miRNAs and endogenous siRNAs in maternal-to-zygotic reprogramming and the establishment of pluripotency. EMBO Rep..

[11] Shin S.W., Tokoro M., Nishikawa S., Lee H.H., Hatanaka Y., Nishihara T., Amano T., Anzai M., Kato H., Mitani T. (2010). Inhibition of the ubiquitin-proteasome system leads to delay of the onset of ZGA gene expression. J. Reprod. Dev..

[12] Tsai T.C., Lin W., Yang S.H., Cheng W.T., Cheng E.H., Lee M.S., Chong K.Y., Chen C.M. (2010). Granzyme G is expressed in the two-cell stage mouse embryo and is required for the maternal-zygotic transition. BMC Dev. Biol..

[13] Galoczova M., Coates P., Vojtesek B. (2018). STAT3, stem cells, cancer cells and p63. Cell. Mol. Biol. Lett..

[14] Raz R., Lee C.K., Cannizzaro L.A., d’Eustachio P., Levy D.E. (1999). Essential role of STAT3 for embryonic stem cell pluripotency. Proc. Natl. Acad. Sci. USA.

[15] Cartwright P., McLean C., Sheppard A., Rivett D., Jones K., Dalton S. (2005). LIF/STAT3 controls ES cell self-renewal and pluripotency by a Myc-dependent mechanism. Development.

[16] Chen C.Y., Lee D.S., Yan Y.T., Shen C.N., Hwang S.M., Lee S.T., Hsieh P.C.H. (2015). Bcl3 bridges LIF-STAT3 to Oct4 signaling in the maintenance of naïve pluripotency. Stem Cells.

[17] Torres J., Watt F.M. (2008). Nanog maintains pluripotency of mouse embryonic stem cells by inhibiting NFkappaB and cooperating with Stat3. Nat. Cell Biol..

[18] Ueda A., Akagi T., Yokota T. (2017). GA-binding protein alpha is involved in the survival of mouse embryonic stem cells. Stem Cells.

[19] Virbasius J.V., Scarpulla R.C. (1991). Transcriptional activation through ETS domain binding-sites in the cytochrome-C-oxidase subunit-IV gene. Mol. Cell. Biol..

[20] Kinoshita K., Ura H., Akagi T., Usuda M., Koide H., Yokota T. (2007). GABP alpha regulates Oct-3/4 expression in mouse embryonic stem cells. Biochem. Biophys. Res. Commun..

[21] Kotch L.E., Iyer N.V., Laughner E., Semenza G.L. (1999). Defective vascularization of HIF-1alpha-null embryos is not associated with VEGF deficiency but with mesenchymal cell death. Dev. Biol..

[22] Fujiwara Y., Browne C.P., Cunniff K., Goff S.C., Orkin S.H. (1996). Arrested development of embryonic red cell precursors in mouse embryos lacking transcription factor GATA-1. Proc. Natl. Acad. Sci. USA.

[23] Dupont S., Krust A., Gansmuller A., Dierich A., Chambon P., Mark M. (2000). Effect of single and compound knockouts of estrogen receptors alpha (ERalpha) and beta (ERbeta) on mouse reproductive phenotypes. Development.

[24] Shivdasani R.A., Mayer E.L., Orkin S.H. (1995). Absence of blood formation in mice lacking the T-cell leukaemia oncoprotein tal-1/SCL. Nature.

[25] Kozak K.R., Abbott B., Hankinson O. (1997). ARNT-deficient mice and placental differentiation. Dev. Biol..

[26] Hoyt P.R., Bartholomew C., Davis A.J., Yutzey K., Gamer L.W., Potter S.S., Ihle J.N., Mucenski M.L. (1997). The Evi1 proto-oncogene is required at midgestation for neural, heart, and paraxial mesenchyme development. Mech. Dev..

[27] Sawada A., Kiyonari H., Ukita K., Nishioka N., Imuta Y., Sasaki H. (2008). Redundant roles of Tead1 and Tead2 in notochord development and the regulation of cell proliferation and survival. Mol. Cell. Biol..

[28] Cummings K.J., Li A., Deneris E.S., Nattie E.E. (2010). Bradycardia in serotonin-deficient Pet-1^−/−^ mice: Influence of respiratory dysfunction and hyperthermia over the first 2 postnatal weeks. Am. J. Physiol. Regul. Integr. Comp. Physiol..

[29] Wang T., Tamakoshi T., Uezato T., Shu F., Kanzaki-Kato N., Fu Y., Koseki H., Yoshida N., Sugiyama T., Miura N. (2003). Forkhead transcription factor Foxf2 (LUN)-deficient mice exhibit abnormal development of secondary palate. Dev. Biol..

[30] Ristevski S., O’Leary D.A., Thornell A.P., Owen M.J., Kola I., Hertzog P.J. (2004). The ETS transcription factor GABPalpha is essential for early embryogenesis. Mol. Cell. Biol..

[31] Braun T., Arnold H.H. (1995). Inactivation of Myf-6 and Myf-5 genes in mice leads to alterations in skeletal muscle development. EMBO J..

[32] Barrionuevo F., Bagheri-Fam S., Klattig J., Kist R., Taketo M.M., Englert C., Scherer G. (2006). Homozygous inactivation of Sox9 causes complete XY sex reversal in mice. Biol. Reprod..

[33] Takeda K., Noguchi K., Shi W., Tanaka T., Matsumoto M., Yoshida N., Kishimoto T., Akira S. (1997). Targeted disruption of the mouse Stat3 gene leads to early embryonic lethality. Proc. Natl. Acad. Sci. USA.

[34] Do D.V., Ueda J., Messerschmidt D.M., Lorthongpanich C., Zhou Y., Feng B., Guo G., Lin P.J., Hossain M.Z., Zhang W. (2013). A genetic and developmental pathway from STAT3 to the OCT4-NANOG circuit is essential for maintenance of ICM lineages in vivo. Genes Dev..

[35] Majumder S., Miranda M., DePamphilis M.L. (1993). Analysis of gene expression in mouse preimplantation embryos demonstrates that the primary role of enhancers is to relieve repression of promoters. EMBO J..

[36] Martinez-Salas E., Linney E., Hassell J., DePamphilis M.L. (1989). The need for enhancers in gene expression first appears during mouse development with formation of the zygotic nucleus. Genes Dev..

[37] Wiekowski M., Miranda M., DePamphilis M.L. (1993). Requirements for promoter activity in mouse oocytes and embryos distinguish paternal pronuclei from maternal and zygotic nuclei. Dev. Biol..

[38] Henery C.C., Miranda M., Wiekowski M., Wilmut I., DePamphilis M.L. (1995). Repression of gene expression at the beginning of mouse development. Dev. Biol..

[39] Rastelli L., Robinson K., Xu Y., Majumder S. (2001). Reconstitution of enhancer function in paternal pronuclei of one-cell mouse embryos. Mol. Cell. Biol..

[40] O’Leary D.A., Noakes P.G., Lavidis N.A., Kola I., Hertzog P.J., Ristevski S. (2007). Targeting of the ETS factor GABPalpha disrupts neuromuscular junction synaptic function. Mol. Cell. Biol..

[41] Yang Z.F., Mott S., Rosmarin A.G. (2007). The Ets transcription factor GABP is required for cell-cycle progression. Nat. Cell Biol..

[42] Yu S., Zhao D.M., Jothi R., Xue H.H. (2010). Critical requirement of GABPalpha for normal T cell development. J. Biol. Chem..

[43] Garcia-Sanz J.A., MacDonald H.R., Jenne D.E., Tschopp J., Nabholz M. (1990). Cell specificity of granzyme gene expression. J. Immunol..

[44] Xue H.H., Bollenbacher-Reilley J., Wu Z., Spolski R., Jing X., Zhang Y.C., McCoy J.P., Leonard W.J. (2007). The transcription factor GABP is a critical regulator of B lymphocyte development. Immunity.

[45] Lim C.P., Cao X. (2006). Structure, function, and regulation of STAT proteins. Mol. BioSyst..

[46] Kamakura S., Oishi K., Yoshimatsu T., Nakafuku M., Masuyama N., Gotoh Y. (2004). Hes binding to STAT3 mediates crosstalk between Notch and JAK-STAT signalling. Nat. Cell Biol..

[47] Yuan Z.L., Guan Y.J., Wang L., Wei W., Kane A.B., Chin Y.E. (2004). Central role of the threonine residue within the p+1 loop of receptor tyrosine kinase in STAT3 constitutive phosphorylation in metastatic cancer cells. Mol. Cell. Biol..

[48] Niwa H., Ogawa K., Shimosato D., Adachi K. (2009). A parallel circuit of LIF signalling pathways maintains pluripotency of mouse ES cells. Nature.

[49] Tang Y., Luo Y., Jiang Z., Ma Y., Lin C.J., Kim C., Carter M.G., Amano T., Park J., Kish S. (2012). Jak/Stat3 signaling promotes somatic cell reprogramming by epigenetic regulation. Stem Cells.

[50] Hellman L.M., Fried M.G. (2007). Electrophoretic mobility shift assay (EMSA) for detecting protein-nucleic acid interactions. Nat. Protoc..

[51] Siddiquee K., Zhang S., Guida W.C., Blaskovich M.A., Greedy B., Lawrence H.R., Yip M.L., Jove R., McLaughlin M.M., Lawrence N.J. (2007). Selective chemical probe inhibitor of Stat3, identified through structure-based virtual screening, induces antitumor activity. Proc. Natl. Acad. Sci. USA.

[52] Ferrajoli A., Faderl S., Van Q., Koch P., Harris D., Liu Z., Hazan-Halevy I., Wang Y., Kantarjian H.M., Priebe W. (2007). WP1066 disrupts Janus kinase-2 and induces caspase-dependent apoptosis in acute myelogenous leukemia cells. Cancer Res..

[53] Laurence A., Pesu M., Silvennoinen O., O’Shea J. (2012). JAK kinases in health and disease: An update. Open Rheumatol. J..

[54] Neubauer H., Cumano A., Muller M., Wu H., Huffstadt U., Pfeffer K. (1998). Jak2 deficiency defines an essential developmental checkpoint in definitive hematopoiesis. Cell.

[55] Griffiths D.S., Li J., Dawson M.A., Trotter M.W.B., Cheng Y.H., Smith A.M., Mansfield W., Liu P., Kouzarides T., Nichols J. (2011). LIF-independent JAK signalling to chromatin in embryonic stem cells uncovered from an adult stem cell disease. Nat. Cell Biol..

[56] Boyman O., Sprent J. (2012). The role of interleukin-2 during homeostasis and activation of the immune system. Nat. Rev. Immunol..

[57] Nielsen M., Nordahl M., Svejgaard A., Odum N. (1998). Interleukin 2 and 15 activate Stat3alpha in human T lymphocytes. Cytokine.

[58] Nielsen M., Kaltoft K., Nordahl M., Röpke C., Geisler C., Mustelin T., Dobson P., Svejgaard A., Odum N. (1997). Constitutive activation of a slowly migrating isoform of Stat3 in mycosis fungoides: Tyrphostin AG490 inhibits Stat3 activation and growth of mycosis fungoides tumor cell lines. Proc. Natl. Acad. Sci. USA.

[59] Pipkin M.E., Sacks J.A., Cruz-Guilloty F., Lichtenheld M.G., Bevan M.J., Rao A. (2010). Interleukin-2 and inflammation induce distinct transcriptional programs that promote the differentiation of effector cytolytic T cells. Immunity.

[60] DeBlaker-Hohe D.A.F., Yamauchi A., Yu C.R., Horvath-Arcidiacono J.A., Bloom E.T. (1995). IL-12 synergizes with IL-2 to induce lymphokine-activated cytotoxicity and perforin and granzyme gene expression in fresh human NK cells. Cell. Immunol..

[61] Kortylewski M., Xin H., Kujawski M., Lee H., Liu Y., Harris T., Drake C., Pardoll D., Yu H. (2009). Regulation of the IL-23 and IL-12 balance by Stat3 signaling in the tumor microenvironment. Cancer Cell.

[62] Allen M.P., Nilsen-Hamilton M. (1998). Granzymes D, E, F, and G are regulated through pregnancy and by IL-2 and IL-15 in granulated metrial gland cells. J. Immunol..

[63] Ostanin A.A., Aizikovich B.I., Aizikovich I.V., Kozhin A.Y., Chernykh E.R. (2007). Role of cytokines in the regulation of reproductive function. Bull. Exp. Biol. Med..

[64] Spanou S., Kalogiannis D., Zapanti E., Gazouli M., Sfontouris I.A., Siristatidis C., Mastorakos G. (2018). Interleukin 15 concentrations in follicular fluid and their effect on oocyte maturation in subfertile women undergoing intracytoplasmic sperm injection. J. Assist. Reprod. Genet..

[65] Robker R.L., Watson L.N., Robertson S.A., Dunning K.R., McLaughlin E.A., Russell D.L. (2014). Identification of sites of STAT3 action in the female reproductive tract through conditional gene deletion. PLoS ONE.

[66] Haraguchi S., Ikeda M., Akagi S., Hirao Y. (2020). Dynamic changes in pStat3 are involved in meiotic spindle assembly in mouse oocytes. Int. J. Mol. Sci..

[67] Guo J., Zhang T., Guo Y., Sun T., Li H., Zhang X., Yin H., Cao G., Yin Y., Wang H. (2018). Oocyte stage-specific effects of MTOR determine granulosa cell fate and oocyte quality in mice. Proc. Natl. Acad. Sci. USA.

[68] Tscherner A., Brown A.C., Stalker L., Kao J., Dufort I., Sirard M.A., LaMarre J. (2018). STAT3 signaling stimulates miR-21 expression in bovine cumulus cells during in vitro oocyte maturation. Sci. Rep..

[69] Zhang L., Badgwell D.B., Bevers J.J., Schlessinger K., Murray P.J., Levy D.E., Watowich S.S. (2006). IL-6 signaling via the STAT3/SOCS3 pathway: Functional analysis of the conserved STAT3 N-domain. Mol. Cell. Biochem..

[70] Zhang X., Darnell J.E. (2001). Functional importance of Stat3 tetramerization in activation of the alpha 2-macroglobulin gene. J. Biol. Chem..

[71] Hu T., Yeh J.E., Pinello L., Jacob J., Chakravarthy S., Yuan G.C., Chopra R., Frank D.A. (2015). Impact of the N-terminal domain of STAT3 in STAT3-dependent transcriptional activity. Mol. Cell. Biol..

[72] Vogt M., Domoszlai T., Kleshchanok D., Lehmann S., Schmitt A., Poli V., Richtering W., Müller-Newen G. (2011). The role of the N-terminal domain in dimerization and nucleocytoplasmic shuttling of latent STAT3. J. Cell Sci..

[73] Martincuks A., Fahrenkamp D., Haan S., Herrmann A., Kuster A., Muller-Newen G. (2016). Dissecting functions of the N-terminal domain and GAS-site recognition in STAT3 nuclear trafficking. Cell Signal..

[74] Iwamaru A., Szymanski S., Iwado E., Aoki H., Yokoyama T., Fokt I., Hess K., Conrad C., Madden T., Sawaya R. (2007). A novel inhibitor of the STAT3 pathway induces apoptosis in malignant glioma cells both in vitro and in vivo. Oncogene.

