# Supplementary material for: STAT3 Is an Upstream Regulator of Granzyme G in the Maternal-To-Zygotic Transition of Mouse Embryos
Source: Int J Mol Sci. 2021 Jan 5;22(1):460. doi: 10.3390/ijms22010460 (PMC7796490; doi:10.3390/ijms22010460)
Supplement: Supplementary file 1 [file ijms-22-00460-s001.pdf]

Supplementary Table S1. The designed primer information for PCR amplification of the *granzyme g* promoter deletion constructs in this study

**Primer sets[1]**	**Oligonucleotide sequence (5′-to-3′)**	**Tm (°C)**
Gzmg promoter FL_F	CGACATGTGAATTTACAACCAGAGTCAT	58
Gzmg promoter FL_R	TACTCGAGGAGGGCAGAGCAGACAC	60
Gzmg promoter Δ1_F	ACATGTAAGAATGGGCTACATGACTAAT	60
Gzmg promoter Δ2_F	ACATGTGCAATACTGTAGGAAATACCAC	60
Gzmg promoter Δ3_F	ACATGTCAGAGATAAATGACAATACCCA	60
Gzmg promoter Δ4_F	ACATGTTACTGTGGAACTTTGAGAGTG	60
ΔStat3mut_F	TACTTGTGTGCAGCCACTGTCTCAGACCTC CTCAACCACAACC	84.9
ΔStat3mut_R	GGTTGTGGTTGAGGAGGTCTGAGACAGTG GCTGCACACAAGTA	84.9

[1]F: forward primer; R: reverse primer

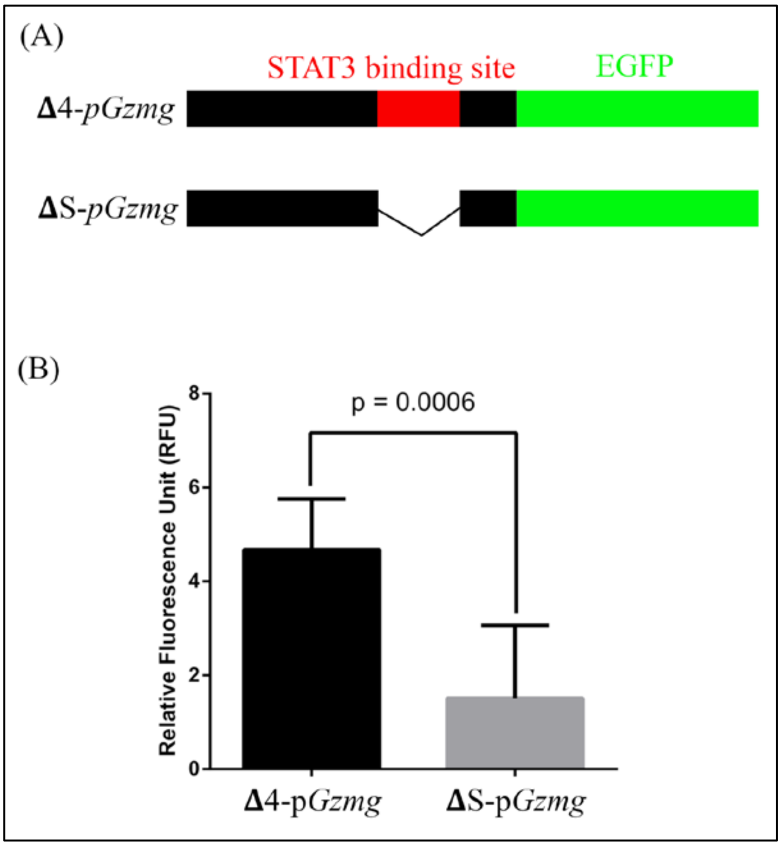

Supplementary Figure S1. The transcription activity of *Gzmg* promoter assay between **Δ**4-p*Gzmg* and **Δ**S-p*Gzmg* in the mouse zygote stage embryos. (**A**) The **Δ**S-*pGzmg* plasmid was constructed by a STAT3 binding site deletion from **Δ**4-*pGzmg* using the site-direct mutagenesis. (**B**) Quantitative data of the *Gzmg* promoter assay obtained by the dual fluorescence system. The graph shows the means ± SD of at least four replicates for each group of embryos. The *p* value (p = 0.0006) indicates a significant difference as determined by Student's t-test ($p < 0.001$).

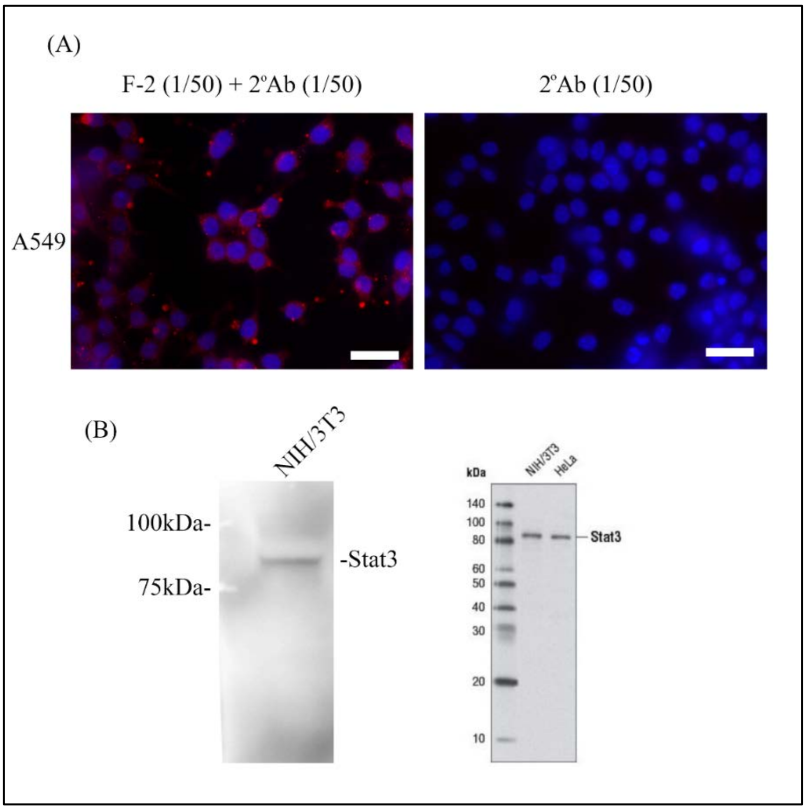

Supplementary Figure S2. Specificity test of F-2 monoclonal antibody in the recognition of STAT3 protein. (**A**) Immunofluorescence staining data showed a specific STAT3 signal in the cell cytoplasm and nucleus of A549 cell was recognized by the F-2 antibody compared with a donkey anti-mouse 2oAb (Alexa546; 1/50 dilution) alone group. Nucleus DNA was stained with DAPI to define nuclear localization. Scale bar = 20 µm. (**B**) Western blot analysis of F-2 monoclonal antibody specificity for STAT3 protein identification using NIH/3T3 mouse embryonic fibroblast cells. (Left panel) The specific signal of STAT3 recognized by F-2 antibody (1/250 dilution) in Western blot analysis of NIH/3T3 cells. (Right panel) The Stat3 Western blot image is from commercial data sheet for F-2 antibody quality control.
